# Association of serological markers with endoscopic mucosal healing and disease activity in patients with inflammatory bowel disease: a retrospective study

**DOI:** 10.3389/fimmu.2026.1830627

**Published:** 2026-07-20

**Authors:** Lu Zhou, Jinfu Dong, Yifan Chen, Yuying Zhou, Zhu Jin, Tianyi Xi, Wen Lu, Henghua Zhou

**Affiliations:** 1 Department of Digestive Endoscopy, Tongren Hospital, Shanghai Jiaotong University School of Medicine, Shanghai, China; 2 Department of General Surgery, The Affiliated Suzhou Hospital of Nanjing Medical University, Suzhou Municipal Hospital, Gusu School Nanjing Medical University, Suzhou, China; 3 Department of General Surgery, Yinshan Lake Hospital, Suzhou, China; 4 Department of Pathology, Tongren Hospital, Shanghai Jiaotong University School of Medicine, Shanghai, China

**Keywords:** Crohn’s disease, disease activity, inflammatory bowel disease, mucosal healing, ulcerative colitis

## Abstract

**Objective:**

This study aimed to examine the link between serological markers and endoscopic mucosal healing and inflammation in diagnosis of inflammatory bowel disease (IBD) patients.

**Methods:**

Clinical data from 200 IBD patients (91 with ulcerative colitis [UC] and 109 with Crohn’s disease [CD]) admitted between December 2022 and June 2024 were retrospectively analyzed, alongside 100 healthy controls. Using the Mayo Endoscopic Score (MES) as the standard, UC patients were divided into an inactive group (n=23) and an active group (n=68). Based on the Simplified Endoscopic Score for Crohn’s Disease (SES-CD), patients in the CD group were divided into an inactive group (n=28) and an active group (n=81). Levels of CRP, TNF-α, ESR, PLT, and ALB were compared. ROC curves assessed predictive efficacy, and Spearman correlation analysis examined correlations.

**Results:**

CRP, TNF-α, ESR, and PLT levels were significantly elevated, but ALB levels were reduced in both UC and CD groups compared to the control group (*p* < 0.05). In inactive patients, CRP, TNF-α, ESR, and PLT levels were lower, but ALB levels were higher compared to active patients in both UC and CD groups (*p* < 0.05). For UC patients, the AUCs for CRP, TNF-α, ESR, PLT, and ALB in assessing mucosal healing were 0.825, 0.712, 0.810, 0.770, and 0.769, respectively, with a combined test AUC of 0.922, surpassing individual markers (CRP: Z = 2.340, *p* = 0.019; TNF-α: Z = 3.488, *p*<0.001; ESR: Z = 2.197, *p* = 0.028; PLT: Z = 2.683, *p* = 0.007; ALB: Z = 2.026, *p* = 0.043). For CD patients, the AUCs for predicting mucosal healing were 0.805, 0.738, 0.763, 0.804, and 0.723 for CRP, TNF-α, ESR, PLT, and ALB, respectively. The AUC of the combined detection was 0.904, which was higher than that of single detection. In UC and CD patients, CRP, TNF-α, ESR, and PLT levels were positively linked to disease inflammation, while ALB was negatively linked (*p* < 0.001).

**Conclusion:**

Serum levels of CRP, TNF-α, ALB, ESR, and PLT are associated with mucosal healing and inflammation in IBD, suggesting they could serve as biomarkers for these conditions.

## 1 Introduction

Inflammatory bowel disease (IBD) is a common clinical chronic inflammatory disease of the intestinal tract, mainly including ulcerative colitis (UC) and Crohn’s disease (CD) (1). The clinical symptoms of IBD usually manifest as recurrent abdominal pain, diarrhea, and mucous blood stools, which may be accompanied by varying degrees of systemic symptoms, seriously affecting the quality of life of patients (2, 3). Endoscopy plays an indispensable role in the diagnosis, condition assessment, and monitoring of treatment effects in IBD, especially endoscopic mucosal healing, which has gradually become a key indicator of treatment effectiveness and disease control. Patients who achieve mucosal healing often have a reduced risk of disease recurrence and a relatively good long-term prognosis (4, 5). Inflammatory activity manifests itself as damage to the intestinal mucosa. During the active phase, the intestinal mucosa shows a variety of pathological changes, which allow visualize of the severity of intestinal inflammation at that time. Therefore, assessment of inflammatory activity is crucial for therapeutic decision-making in IBD (6).

Fecal calprotectin (FC) is currently recognized as a non-invasive biomarker that strongly correlates with endoscopic activity and has been recommended by multiple guidelines for disease monitoring in IBD (7). However, FC testing requires patients to provide fecal samples. Some patients exhibit poor compliance or improper sample collection, which limits its clinical utility. In contrast, routine serological indicators offer advantages of accessibility, reproducibility, and minimal patient burden (8). However, previous studies have predominantly focused on individual markers, lacking a systematic integration of multiple pathological dimensions. To address this gap, we strategically selected five routine serological parameters based on their established pathophysiological roles and documented correlations with intestinal inflammation and mucosal integrity in IBD. First, C-reactive protein (CRP), an acute-phase reactant, is rapidly elevated in response to intestinal inflammation and has been repeatedly validated as a sensitive indicator of disease activity in both UC and CD (9–11). Second, erythrocyte sedimentation rate (ESR), reflecting plasma protein changes and erythrocyte aggregation, has shown a close association with active IBD and is often used as an adjunct to CRP (12, 13). Third, platelet count (PLT) is included because platelets are not only key players in hemostasis but also contribute to the immune-inflammatory cascade in IBD; their elevation correlates with mucosal injury and has been linked to disease activity in previous reports (14, 15). Fourth, serum albumin (ALB), as a marker of nutritional status and hepatic synthetic function, has been found to be inversely correlated with the severity of intestinal inflammation and mucosal damage in IBD patients, with hypoalbuminemia indicating a poorer healing capacity (16–18). Fifth, tumor necrosis factor-α (TNF-α) represents a core pro-inflammatory cytokine in the IBD inflammatory network; it drives the NF-κB pathway, compromises intestinal epithelial barrier function, and its overexpression has been consistently associated with active disease and failure of mucosal healing (19, 20). Therefore, by covering the acute inflammatory response (CRP, ESR), platelet-mediated immune activation (PLT), nutritional/inflammatory status (ALB), and key cytokine-driven inflammation (TNF-α), this panel was hypothesized to provide a more comprehensive, non-invasive assessment of mucosal healing. The selected indicators are routinely and cost-effectively measured in most clinical settings, potentially offering broader applicability compared to specialized or fecal-based assays.

## 2 Materials and methods

### 2.1 Patients

**Figure 1:**
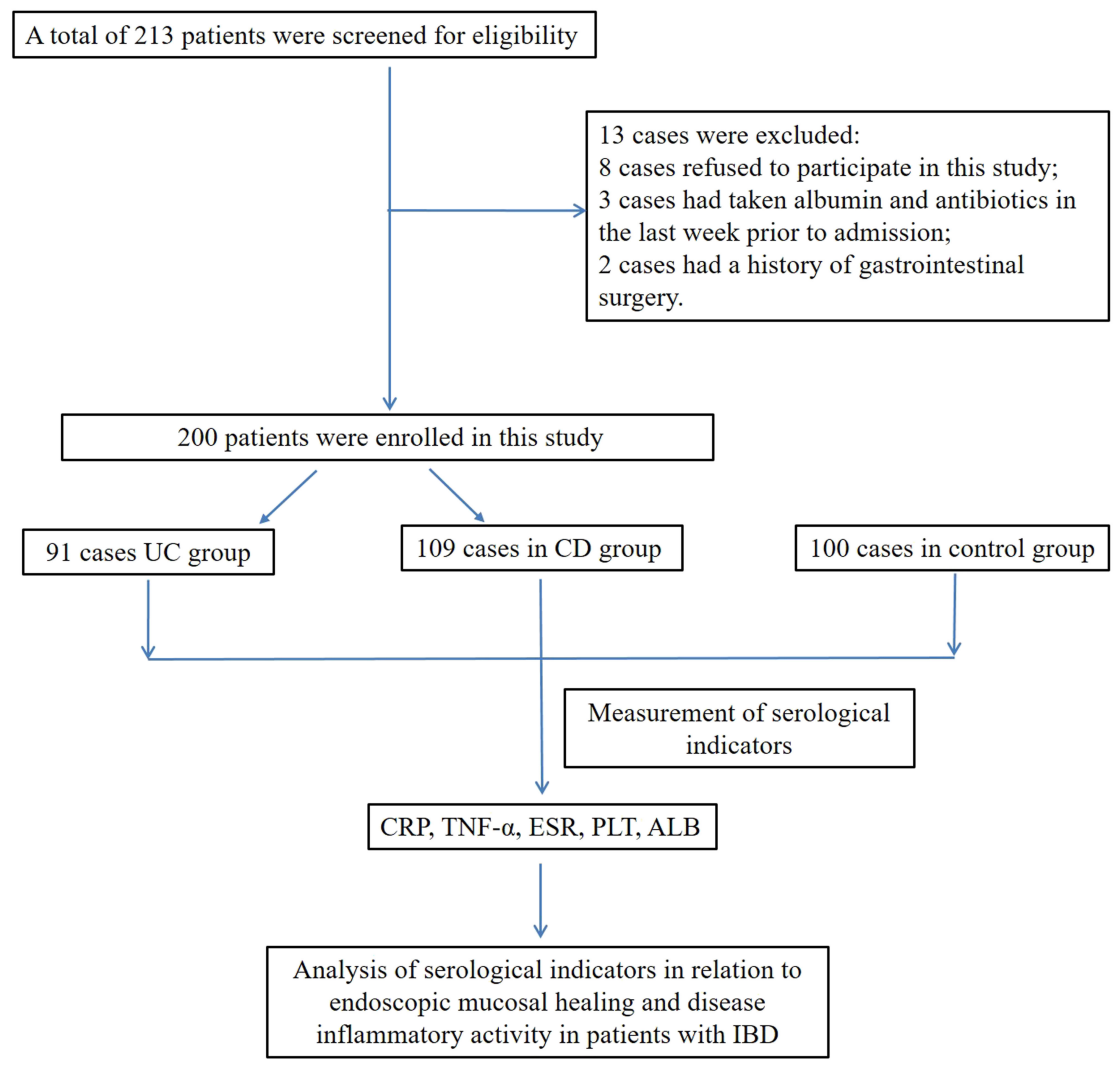
Flow chart.

Patients with IBD admitted to Shanghai Sixth People’s Hospital Affiliated to Shanghai Jiao Tong University School of Medicine from December 2022 to June 2024 were retrospectively selected. Inclusion criteria: (1) meeting the diagnostic criteria for IBD (21); age >18 years. Exclusion criteria: (1) use of albumin, antibiotics, or other drugs affecting serological indices with one week before admission; (2) presence of infectious or autoimmune diseases; (3) combined malignant tumors; (4) history of gastrointestinal surgery; (5) insufficiency of heart, liver, kidneys, or other vital organs. A total of 100 healthy individuals who underwent medical check-ups during the same period were selected as the control group. Inclusion criteria for the control group: (1) normal stool frequency without any gastrointestinal symptoms; (2) no family history of IBD; (3) age >18 years. Exclusion criteria: (1) history of gastrointestinal surgery within the last three months; (2) pregnant or lactating women; (3) history of psychiatric disorders. A total of 200 patients with IBD were finally enrolled in this study (**Figure 1**), including 91 with UC and 109 with CD. This study was approved by the Ethics Committee of the Affiliated Suzhou Hospital of Nanjing Medical University (Approval number: K-2022-096). Written informed consent was obtained from all individual participant included in the study. The clinical data used in this study were obtained from the hospital’s electronic medical record system and biobank. The extraction and use of data were authorized by the hospital’s information management department and the Biobank Management Committee.

### 2.2 Detection of serologic indexes

Fasting peripheral venous blood samples were collected from all patients within 48 hours of admission and before the initiation of any induction therapy (including glucocorticoids, biologics, or immunosuppressants). Platelet counts were measured by the resistive impedance method, and the PLT value was recorded using a fully automated blood cell analyzer (HST-N201, SYSMEX, Kobe, Japan). Serum CRP and ALB levels were measured by and immunoturbidimetric method and the bromocresol green method, respectively, using a BC-7500 automatic biochemical analyzer (Mindray, Shenzhen, China). ESR was determined using an SD-1000 automated ESR analyzer (Beijing Succeede, Beijing, China). Serum TNF-α levels were measured using an enzyme-linked immunosorbent assay kit (ab181421, Abcam, Cambridge, MA, USA).

### 2.3 Endoscopic scoring

On the same day as blood collection, all patients underwent total colonoscopy (for UC patients) or ileocolonoscopy (for CD patients), which were independently performed and scored by two experienced endoscopists who were blinded to the patients’ serological results. In cases of scoring disagreement, the two physicians reached a consensus through discussion. (1) UC: Endoscopic disease activity in UC patients was assessed using the Mayo Endoscopic Score (MES) (22). A score of 0 indicated no active inflammation, 1 indicated mild activity, 2 moderate activity, and 3 severe activity. Based on the MES, UC patients were classified into the “inactive” group (n=23), mild group (n=25), moderate group (n=31), and severe group (n=12). It should be noted that this study adopted a cross-sectional design, assessing endoscopic status at a single time point; therefore, the term “inactive” is used instead of “healing, ” as the latter requires follow-up comparison after treatment. (2) CD: Endoscopic disease activity in CD patients was assessed using the Simplified Endoscopic Score for Crohn’s Disease (SES-CD) (23). An SES-CD score of 0 indicated no endoscopic active inflammation, 1–3 indicated mild activity, 4–7 indicated moderate activity, and 8–12 indicated severe activity. Based on the SES-CD, CD patients were classified into the “inactive” group (n=28), mild group (n=27), moderate group (n=39), and severe group (n=15).

### 2.4 Statistical analysis

Statistical analyses were performed using IBM SPSS Statistics (Version 16.0. Armonk, NY, USA: IBM Corp), with *p* values < 0.05 indicating statistically significance. The proportions of categorical variables were compared using Chi-square test or Fisher’s exact test. Continuous variables were presented as mean and standard deviation or median and range. Continuous variables were compared using the independent two-sample t-test or the Mann–Whitney U test, as appropriate. Spearman’s rank correlation analysis was used to assess the correlation between serologic indices and disease activity in IBD patients. The value of each serological index, alone or in combination, for assessing endoscopic inactivity (or “inactive” status) in IBD patients was analyzed using receiver operating characteristic (ROC) curves.

## 3 Results

### 3.1 General information

There was no significant difference in gender distribution and age among the CD, UC and control groups (*p* > 0.05, **Table 1**). There was no significant difference in endoscopic healing between the CD and UC groups (*p* > 0.05, **Table 1**).

**Table 1:** Comparison of the clinical characteristics of the three groups.

Parameters	UC group (n=91)	CD group (n=109)	Control group (n=100)	*F*/*χ*^2^	*P*
Sex [n (%)]				0.082	0.960
Male	50 (54.95)	61 (55.96)	57 (57.00)		
Female	41 (45.05)	48 (44.04)	43 (43.00)		
Age (years)	34.78 ± 5.05	36.25 ± 5.39	34.89 ± 5.88	2.331	0.099
Extent of lesion [n (%)				–	–
Terminal ileum	–	14 (12.84)	–		
Colon	–	7 (6.42)	–		
Ileum	–	75 (68.81)	–		
Upper gastrointestinal tract	–	3 (2.74)	–		
Extent of lesion [n (%)				–	–
Rectum	15 (16.48)	–	–		
Left hemicolon	20 (21.98)	–	–		
Whole colon	56 (61.54)	–	–		
Endoscopic condition [n (%)				0.196	0.995
Healing	23 (25.27)	28 (25.69)	–		
Mild	25 (27.47)	27 (24.77)	–		
Moderate	31 (34.07)	39 (35.78)	–		
Severe	12 (13.19)	15 (13.76)	–		

### 3.2 Comparison of serologic index levels between IBD patients and controls

**Figure 2:**
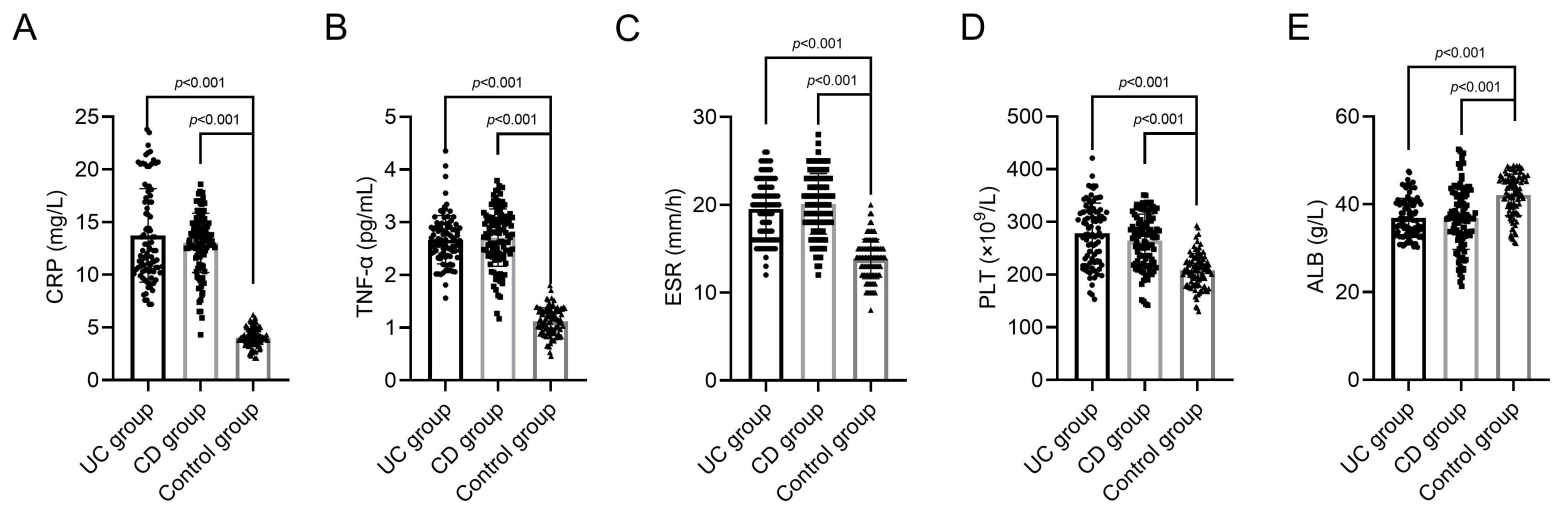
Comparison of serologic index levels between IBD patients and controls. **(A)** The levels of CRP among UC, CD and Control groups. **(B)** The levels of TNF-α among UC, CD and Control groups, **(C)** The levels of ESR among UC, CD and Control groups. **(D)** The levels of PLT among UC, CD and Control groups. **(E)** The levels of ALB among UC, CD and Control groups. UC group (n=91), CD group (n=109) and Control group (n=100).

Compared with the control group, patients in the UC and CD groups had significantly higher CRP (**Figure 2A**), TNF-α (**Figure 2B**), ESR (**Figure 2C**), and PLT (**Figure 2D**) levels (*p* < 0.05), while ALB levels (**Figure 2E**) were significantly lower (*p* < 0.05). There was no significant difference in the levels of CRP, TNF-α, ESR, PLT and ALB between the UC and CD groups (*p* > 0.05, **Figure 2**).

### 3.3 Comparison of serum parameters in patients with IBD between inactive group and active group

Based on endoscopic disease activity, patients in the UC and CD groups were further divided into inactive groups (UC: MES ≤ 1, n=23; CD: SES-CD ≤ 2, n=28) and active groups (UC: MES ≥ 3, n=68; CD: SES-CD ≥ 5, n=81).

**Figure 3:**
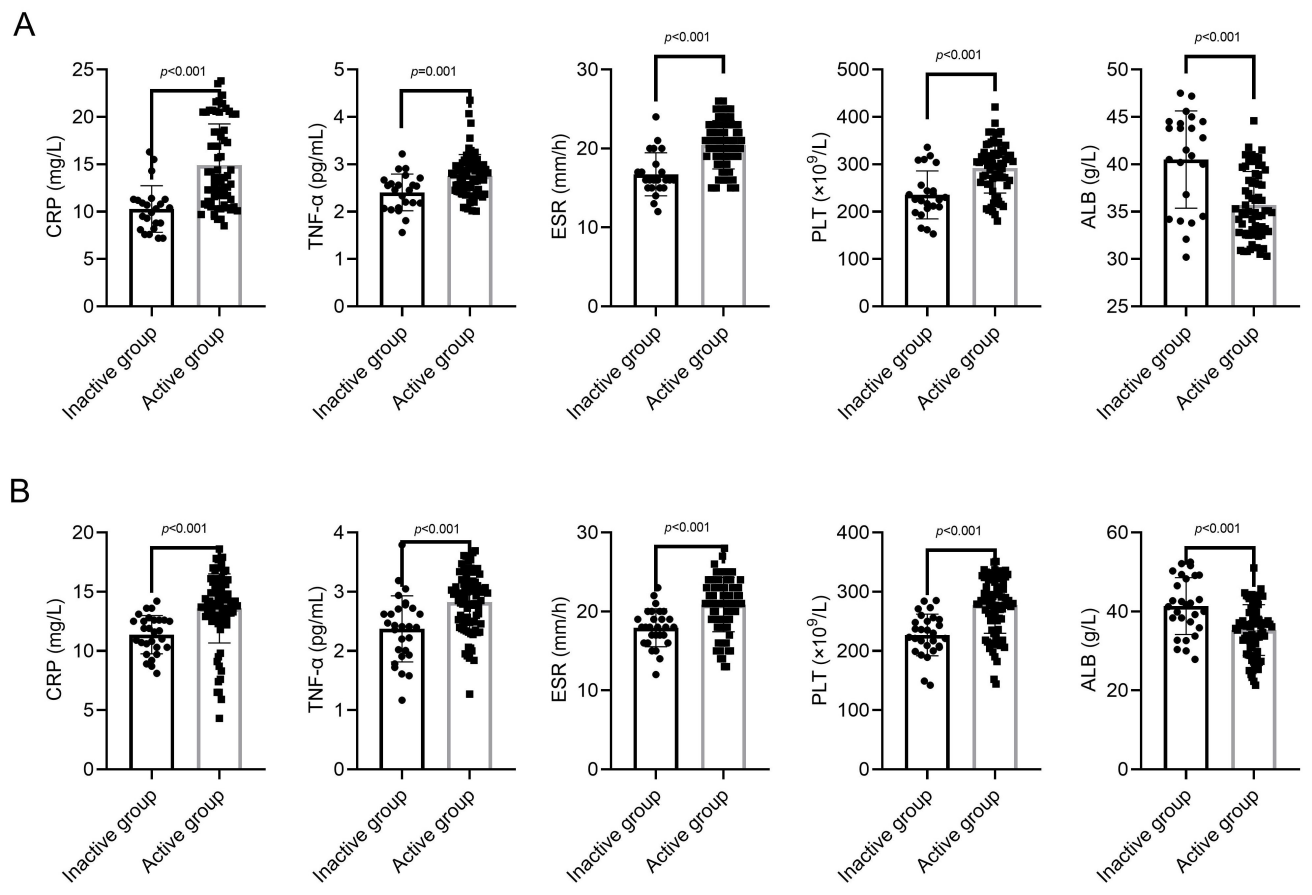
Comparison of serum indicators between active group (n=68) and inactive group in patients with IBD. **(A)** The levels of CRP, TNF-α, ESR, PLT and ALB between active group (n=68) and inactive group (n=23) in UC patients. **(B)** The levels of CRP, TNF-α, ESR, PLT and ALB between active group (n=81) and inactive group (n=28) in CD patients.

Compared with the active group, the levels of CRP, TNF-α, ESR, and PLT were significantly lower in inactive groups in both the UC (**Figure 3A**) and CD (**Figure 3B**, *p* < 0.05), while ALB levels were significantly higher in both groups (*p* < 0.05; **Figures 3A, B**).

### 3.4 Performance of serum biomarkers in identifying endoscopic inactivity in patients with IBD

**Figure 4:**
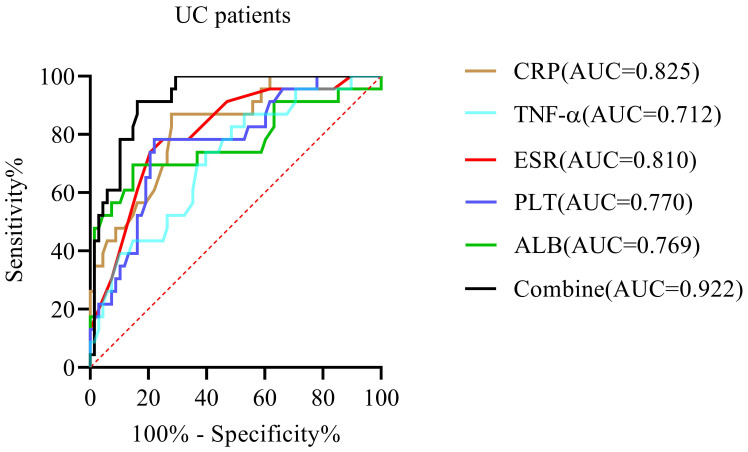
ROC curve of serum indicators to assess mucosal healing in patients with UC.

The AUCs of CRP, TNF-α, ESR, PLT, and ALB for predicting endoscopic inactive inflammation in patients with UC were 0.825, 0.712, 0.810, 0.770, and 0.769 respectively (**Table 2**, **Figure 4**). A binary logistic regression model was used to build a joint probability model for serum indicators to jointly predict endoscopic inactive inflammation in patients with UC, and the joint prediction probability (P) for each patient was calculated. This prediction probability was used as a new joint predictor to draw an ROC curve. The results showed that the AUC of the combination of these five indicators was 0.922, which was higher than that of CRP (Z = 2.340, *p* = 0.019), TNF-α (Z = 3.488, *p* <.001), ESR (Z = 2.197, *P* = 0.028), PLT (Z = 2.683, *p* = 0.007), and ALB (Z = 2.026, *P* = 0.043), respectively (**Table 2**, **Figure 4**). The maximum Youden index method was used to determine the optimal cut-off value for the combined predicted probability as 0.18. The clinical implication of this cut-off value is that when the combined predicted probability for a UC patient exceeds 0.18, it suggests a high likelihood of achieving endoscopic inactive inflammation (i.e., mucosal healing). At this cut-off value, the combined prediction yielded a sensitivity of 86.92%, a specificity of 85.29%, and a Youden index of 0.723 (**Table 2**, **Figure 4**).

**Table 2:** Efficacy analysis of serum indexes in judging the absence of active inflammation under endoscopy in UC patients.

Variables	AUC	Cut-off value	95%*CI*	Sensitivity (%)	Specificity (%)	Youden’s index	*P*
CRP	0.825	11.4	0.731-0.897	86.96 (20/23)	72.06 (49/68)	0.590	<0.001
TNF-α	0.712	2.59	0.608-0.802	73.91 (20/23)	60.29 (28/68)	0.342	<0.001
ESR	0.810	17	0.714 -0.884	73.91 (17/23)	79.41 (54/68)	0.533	<0.001
PLT	0.770	256	0.670-0.852	78.26 (18/23)	77.94 (53/68)	0.562	<0.001
ALB	0.769	39.9	0.669-0.851	69.57 (16/23)	85.29 (58/68)	0.549	<0.001
Combine	0.922	0.18	0.847-0.968	86.92 (20/23)	85.29 (58/68)	0.723	<0.001

**Figure 5:**
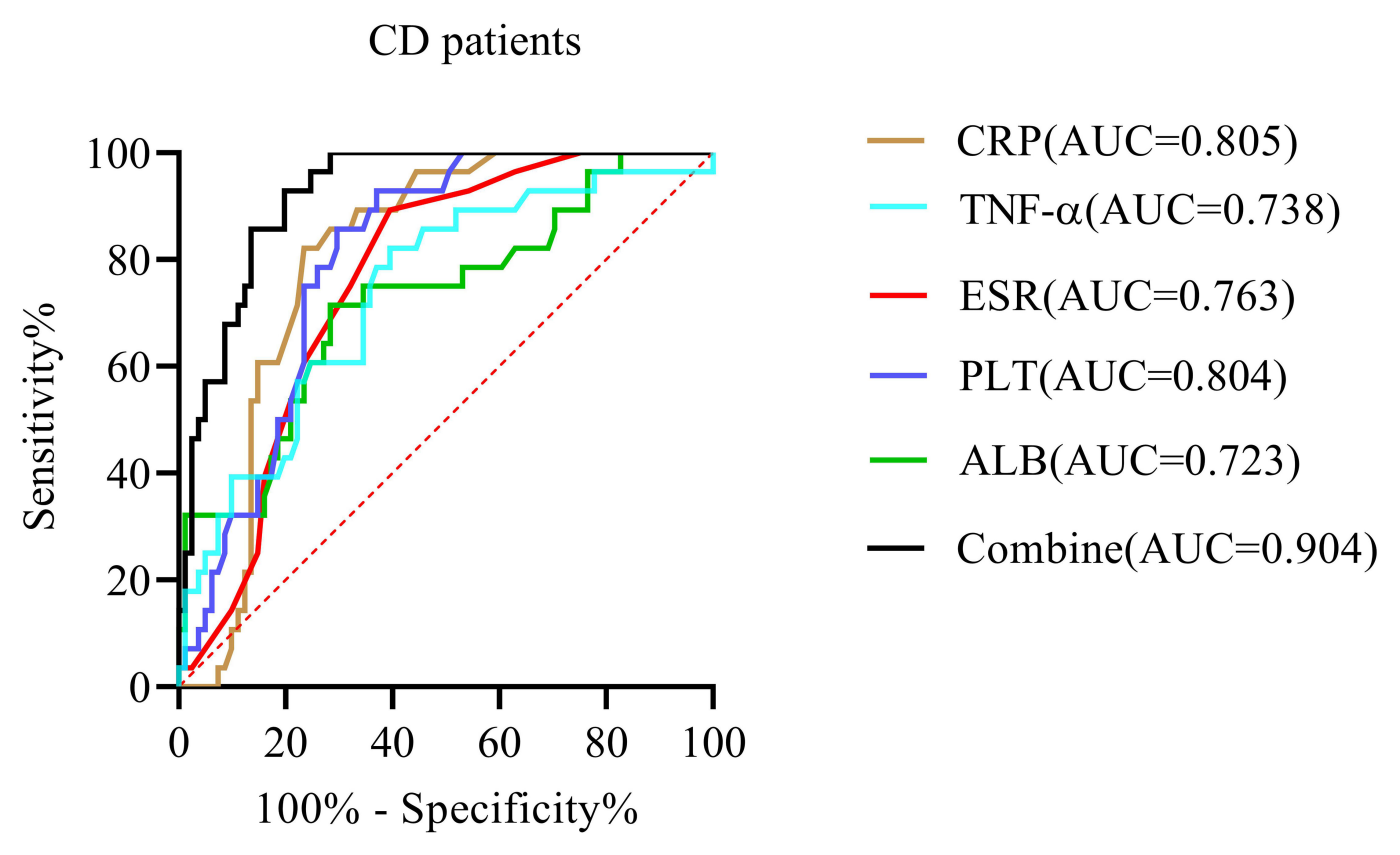
ROC curves of serum indicators to predict mucosal healing in patients with CD.

The AUCs of CRP, TNF-α, ESR, PLT, and ALB for predicting endoscopic inactive inflammation in patients with CD were 0.805, 0.738, 0.763, 0.804, and 0.723, respectively (**Table 3**, **Figure 5**). Similarly, a binary Logistic regression model was used to build a joint probability model for serum indicators to jointly predict endoscopic inactive inflammation in patients with CD. The results showed that when the five serum indicators were combined, the AUC of the combination was 0.904, which was higher than that of CRP (Z = 2.821, *p* = 0.005), TNF-α (Z = 3.850, *p* < 0.001), ESR (Z = 3.752, *p* < 0.001), PLT (Z = 2.882, P = 0.004), and ALB (Z = 3.914, *p* < 0.001), respectively (**Table 3**, **Figure 5**). The maximum Youden index method was used to determine the optimal cut-off value for the combined predicted probability as -0.93. It should be noted that this negative cut-off value results from the combined calculation of the constant term and regression coefficients of individual variables in the logistic regression model; it is essentially a linear combination of the predicted probability after logit transformation. In clinical practice, the combined predicted probability can be calculated by substituting the patient’s serological markers into the above formula. If the calculated value is greater than -0.93, the patients may have endoscopic inactive inflammation. At this cut-off value, the combined prediction yielded a sensitivity of 85.71%, a specificity of 82.72%, and a Youden index of 0.731 (**Table 3**, **Figure 5**).

**Table 3:** Efficacy analysis of serum indexes in judging the absence of active inflammation under endoscopy in CD patients.

Variables	AUC	Cut-off value	95%*CI*	Sensitivity (%)	Specificity (%)	Youden’s index	*P*
CRP	0.805	12.6	0.691-0.867	82.14 (23/28)	76.54 (62/81)	0.546	<0.001
TNF-α	0.738	2.78	0.645-0.817	82.14 (23/28)	60.49 (49/81)	0.426	<0.001
ESR	0.763	20	0.672-0.839	89.29 (25/28)	60.49 (49/81)	0.498	<0.001
PLT	0.804	260	0.700-0.861	85.71 (24/28)	70.37 (57/81)	0.561	<0.001
ALB	0.723	38.0	0.629-0.805	71.43 (20/28)	71.60 (58/81)	0.430	<0.001
Combine	0.904	-0.93	0.833-0.952	85.71 (24/28)	82.72 (67/81)	0.731	<0.001

The Bootstrap method (with 1, 000 resampling iterations) was used for internal validation of the above combined model. The results showed that the bias-corrected AUC for the UC group was 0.906 (95% CI: 0.877–0.929), and for the CD group, it was 0.886 (95% CI: 0.859–0.913). The bias of the corrected model AUC was small, indicating that the combined model in this study has a good fit and a certain potential for generalizability.

### 3.5 Comparison of serum indicators in patients with different levels of inflammatory activity

**Figure 6:**
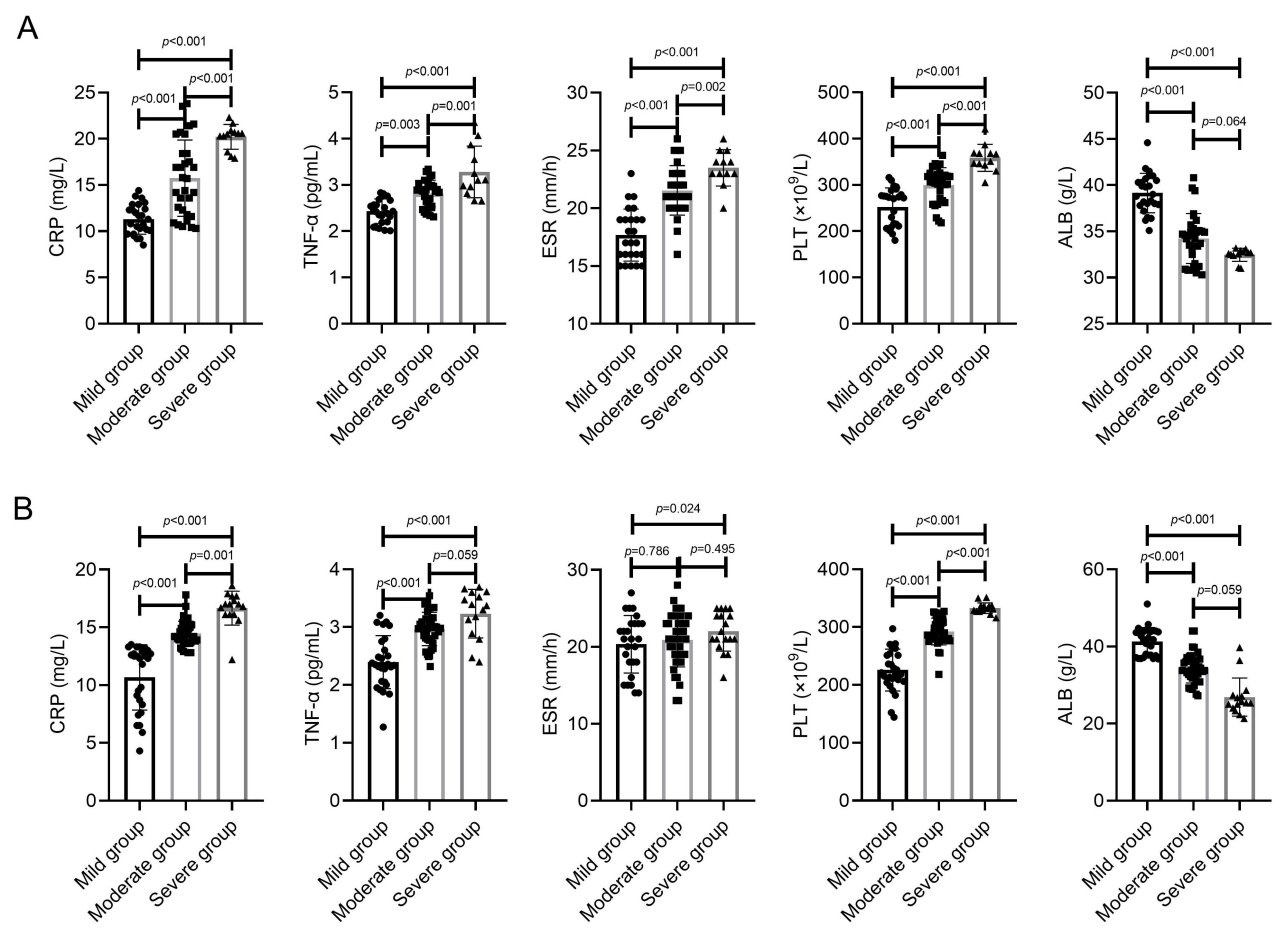
Comparison of serum indicators in patients with different levels of inflammatory activity. **(A)** The levels of CRP, TNF-α, ESR, PLT and ALB among mild (n=25), moderate (n=31) and severe (n=12) groups in UC patients. **(A)** The levels of CRP, TNF-α, ESR, PLT and ALB among mild (n=27), moderate (n=39) and severe (n=15) groups in CD patients.

In active patients with UC, the levels of CRP, TNF-α, ESR, and PLT were progressively increased with worsening of severity (*p* < 0.05, **Figure 6A**). The ALB level in the moderate group was significantly lower than that in the mild group (*p* < 0.05, **Figure 6A**), but no statistically significant difference was observed between the moderate and severe groups (*p* > 0.05, **Figure 6A**). Similarly, in active patients with CD, the levels of CRP and PLT were progressively increased with worsening of severity (*p* < 0.05, **Figure 6B**). In addition, compared with the mild group, the moderate group showed a significantly higher TNF-α level and a significantly lower ALB level (*p* < 0.05, **Figure 6B**). However, no significant differences in TNF-α or ALB levels were found between the moderate and severe groups (*p* > 0.05, **Figure 6B**). Furthermore, although there were no significant differences in ESR levels between the mild and moderate groups or between the moderate and severe groups (*p* > 0.05, **Figure 6B**), the ESR level was differed significantly between the mild and severe groups (*p* < 0.05, **Figure 6B**).

### 3.6 Correlation analysis between serum indicators and inflammatory activity of the disease

The results of Spearman correlation analysis showed that CRP, ESR, and PLT were positively correlated with the degree of inflammatory activity in both the UC and CD groups (*p* < 0.05, **Table 4**), whereas ALB was negatively correlated with the degree of inflammatory activity (*p* < 0.05, **Table 4**).

**Table 4:** Correlation analysis between serum indicators and inflammatory activity of the disease.

Group	Indicators	*r*	*P*
UC group	CRP	0.709	<0.001
	TNF-α	0.659	<0.001
	ESR	0.741	<0.001
	PLT	0.719	<0.001
	ALB	-0.766	<0.001
CD group	CRP	0.833	<0.001
	TNF-α	0.627	<0.001
	ESR	0.797	<0.001
	PLT	0.871	<0.001
	ALB	-0.792	<0.001

## 4 Discussion

UC and CD share epidemiologic features characterized by a chronic, relapsing-remitting, and intermittent course. Although endoscopy remains the gold standard for assessing disease activity, it is limited by invasiveness, high cost, poor patient tolerance, prolonged bowel preparation, and associated complication risks. Therefore, establishing accurate, cost-effective, and accessible indicators of the degree of mucosal healing is of critical importance. Such indicators should also be safe, timely, and relatively simple to apply in the diagnosis and management of patients with IBD.

As objective tests reflecting intestinal disease, immune function, and systemic inflammation, serologic markers have drawn considerable interest (24). In recent years, serologic indicators have shown potential value in the diagnosis, disease assessment, treatment selection, and efficacy monitoring of IBD (24). In this study, we found that CRP, TNF-α, ESR, PLT and ALB all showed significant correlations with endoscopic disease activity in patients with IBD, suggesting that they may serve as non-invasive biomarkers for assessing the mucosal status in IBD. CRP, an acute-phase response protein, is synthesized and released into the circulation by hepatocytes upon stimulation by cytokines such as interleukin- 6 (IL-6) and TNF-α during the inflammatory response (25). In IBD, persistent inflammatory stimulation of the intestinal mucosa leads to elevated CRP levels (10). When intestinal inflammation is poorly controlled and mucosal healing is inadequate, the inflammatory response continues, and CRP synthesis and secretion remain elevated. ESR is an inflammatory marker whose levels rise in response to infection and inflammation; it has similar value to CRP in assessing inflammation. Previous studies have shown that an elevated ESR reflects increased erythrocyte aggregation, which is most often observed in active or relapsing disease and is closely associated with inflammatory conditions (11). PLT plays a crucial role in hemostasis, vascular repair, and immune-inflammatory responses. In the inflammatory state, the hematopoietic microenvironment is altered, and the levels of several cytokines and growth factors are elevated. These factors stimulate bone marrow megakaryocyte proliferation and differentiation, leading to increased platelet production (26). ALB is synthesized in the liver and released into the peripheral circulation, with its metabolism regulated by multiple factors (27). Inflammatory factors (e.g., TNF-α, IL-6) in patients with IBD can act directly on hepatocytes to inhibit ALB synthesis. Meanwhile, intestinal inflammation impairs the mucosal barrier, resulting in increased protein loss from the gut (12). These multifaceted factors ultimately lead to decreased levels of ALB in IBD patients. TNF-α is mainly secreted by immune cells such as activated macrophages, T lymphocytes, and natural killer cells. Under normal conditions, TNF-α participates in host defense and promotes inflammation to clear pathogens. However, in IBD patients, overexpression of TNF-α may lead to persistent intestinal inflammation and tissue damage (20). As reported by Meima-van Praag et al. (20), TNF-α contributes to pathogenesis of IBD by binding to its receptors TNFR1 and TNFR2, which in turn activate a series of downstream signaling pathways, leading to the release of inflammatory mediators and subsequent tissue injury. Therefore, the above serologic indicators are valuable in the diagnosis, identification, and disease evaluation of IBD.

This study also found that the AUCs of CRP, TNF-α, ESR, PLT, and ALB for predicting endoscopic inactive inflammation in patients with UC and CD were all greater than 0.7. When these indices were applied in combination, the AUC exceeded 0.9. These results suggested that serum CRP, ESR, PLT, and ALB levels could be used as valuable serologic indicators for predicting endoscopic activity in IBD, with higher predictive value when applied in combination. The erythrocyte sedimentation rate mainly depends on changes in the ratios of plasma protein components. Increased inflammatory factors in IBD patients elevate plasma components such as fibrinogen and globulin, leading to faster sedimentation (13). Therefore, we hypothesized that monitoring serum ESR could provide a reference for the clinical assessment of IBD patients. Bahaa A et al. (28) reported that the serologic biomarkers IL-6 and CRP showed good clinical value in assessing disease activity in IBD patients, which is consistent with the findings of this study. Different serologic indicators reflect inflammatory state, immune status, and intestinal mucosal repair in IBD from different perspectives. Comprehensive analysis of these indicators enables more accurate disease assessment and improves predictive accuracy.

This study also found that serum CRP, TNF-α, ESR, PLT, and ALB levels were associated with the degree of inflammatory activity in patients with IBD. The severity of intestinal inflammation is related to CRP elevation. In this setting, plasma macromolecules such as globulin also increase more markedly, leading to faster blood sedimentation. Therefore, ESR is positively correlated with inflammatory activity and can assist in determining the severity of intestinal inflammation. Elevated PLT levels may be related to the inflammatory environment in IBD patients and the physiological and functional properties of platelets. Platelets are activated under inflammatory condition. On the one hand, they participate in immune regulation and tissue repair at the site of inflammation. On the other hand, interactions between platelets, vascular endothelial cells, and leukocytes further promote the inflammatory response, ultimately increasing platelet counts in the blood (15, 29). During the active phase of IBD, inflammatory damage to the intestinal mucosa disrupts intestinal barrier function. Consequently, large amounts of inflammatory mediators are released into the bloodstream, triggering a systemic inflammatory response. This process impairs liver metabolic function, redirecting resources toward the synthesis of acute-phase response proteins and reducing ALB synthesis (17). In addition, inflammation-induced increases in metabolic demand accelerate albumin catabolism and decrease blood ALB levels. Under the combined effect of these factors, ALB levels decrease as disease activity increases (18). Therefore, changes in the levels of the above serologic markers are closely associated with disease severity in IBD patients, suggesting that these markers may serve as valuable biomarkers for disease assessment.

Nevertheless, this study has several limitations. First, given the complex pathogenesis of IBD, the parameters included in this study were limited. Second, this study is a single-center exploratory analysis designed to initially evaluate serological markers associated with endoscopic mucosal healing in IBD patients. All cutoff values were determined based on this cohort data. As no independent external cohort was used for validation, the generalizability of our conclusions requires further confirmation in future multicenter, large-sample studies. Third, limited by the number of positive samples (23 UC cases, 28 CD cases), the events-per-variable (EPV) principle precluded multivariate analysis to adjust for potential confounders such as age, disease duration, and treatment. Fourth, due to the retrospective single-center design and lack of an external validation cohort, the reported cutoff values and combined AUC should be interpreted as hypothesis-generating rather than clinically definitive. Fifth, this study does not provide direct experimental evidence of causality; the proposed roles of these markers are inferred from correlation and prior literature. Sixth, FC is a well-recognized non-invasive biomarker in the IBD field and is considered to be the most strongly correlated with intestinal mucosal inflammation. Due to the retrospective nature of this study, FC data were not available. Therefore, it is not possible to directly compare these five serological markers with FC; moreover, we also cannot evaluate the incremental predictive value of the proposed serological test combination relative to fecal calprotectin. Future studies are still needed to compare the serological panel obtained in this study with FC and to conduct prospective dynamic follow-up to more fully assess the clinical application scenarios of serological parameters in IBD management.

In conclusion, serum CRP, ESR, PLT, ALB, and TNF-α levels are associated with endoscopic mucosal healing and disease activity in IBD patients. However, given the retrospective, single-center design, these markers should be considered adjunctive to endoscopy rather than replacements. Prospective, multicenter studies with predefined cutoff values are needed before clinical implementation.

## Data Availability

The original contributions presented in the study are included in the article/supplementary material. Further inquiries can be directed to the corresponding authors.
